# Supplementary material for: Exchange anisotropies in microwave-driven singlet-triplet qubits
Source: Nat Commun. 2025 Apr 24;16:3862. doi: 10.1038/s41467-025-58969-y (PMC12022185; doi:10.1038/s41467-025-58969-y)
Supplement: Supplementary file 1 — Supplementary Information [file 41467_2025_58969_MOESM1_ESM.pdf]

Supplementary Information: Exchange anisotropies in microwave-driven singlet-triplet qubits

Jaime Saez-Mollejo[1], Daniel Jirovec[1,2], Yona Schell[1], Josip Kukucka[1], Stefano Calcaterra[3], Daniel Chrastina[3], Giovanni Isella[3], Maximilian Rimbach-Russ[2], Stefano Bosco[2], and Georgios Katsaros[1]

[1] *Institute of Science and Technology Austria, Klosterneuburg, Austria.*
[2] *QuTech, Delft University of Technology, Delft, The Netherlands. and*
[3] *Laboratory for Epitaxial Nanostructures on Silicon and Spintronics, Physics Department, Politecnico di Milano, Como, Italy.*

CONTENTS

Supplementary Note 1. Experimental setup 2

Supplementary Note 2. Geometrical interpretation 3

Supplementary Note 3. Rabi chevrons and gate fidelities 4

Supplementary Note 4. Raw data of Rabi oscillation dependences 6

Supplementary Note 5. Lever arms and cross-capacitance of gate BR 10

Supplementary Note 6. Extra out-of-plane data 11

Supplementary Note 7. Subharmonic processes 12

Supplementary Note 8. Noise model and fitting parameters 14

Supplementary Note 9. Fits of Ramsey oscillations 15

References 20

SUPPLEMENTARY NOTE 1. EXPERIMENTAL SETUP

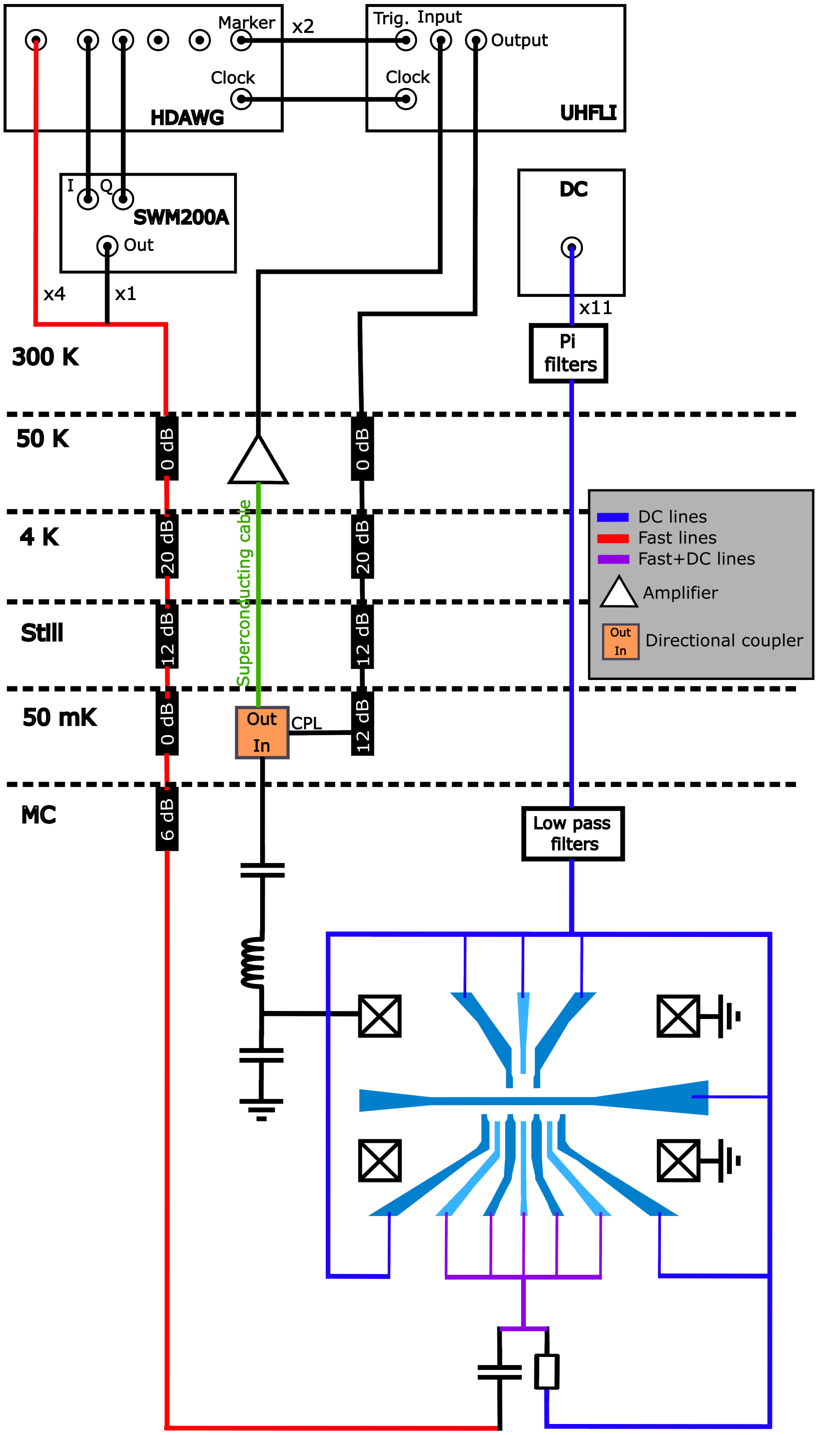

Supplementary Figure 1. **Sketch of experimental setup.** High-frequency lines (red color) are attenuated at the different stages of the cryostat to minimize the electron temperature. The attenuators are represented as black boxes with the corresponding values. A 0 dB attenuator acts as a thermal anchor. The fast lines are connected to an Arbitrary Waveform Generator from Zurich Instruments (HDAWG) to change the charge occupation of the double quantum dot, and one line is connected to a Rhode Schwarz microwave source (SMW200A) to drive the qubit. The DC lines (in blue) are connected to a Delft-IVVI rack. The UHFLI generates and demodulates the microwave reflectometry signal. This reflectometry tone is attenuated and applied to an ohmic contact of the charge sensor with a directional coupler (MiniCircuits ZFDC-20-50 S+). The tank circuit used for reflectometry comprises an inductance (L) of 470 nH and an approximate parasitic capacitance (C_p) of 0.9 pF. The reflected signal is amplified at the 50 K stage by a cryogenic amplifier (CITLF3).

SUPPLEMENTARY NOTE 2. GEOMETRICAL INTERPRETATION

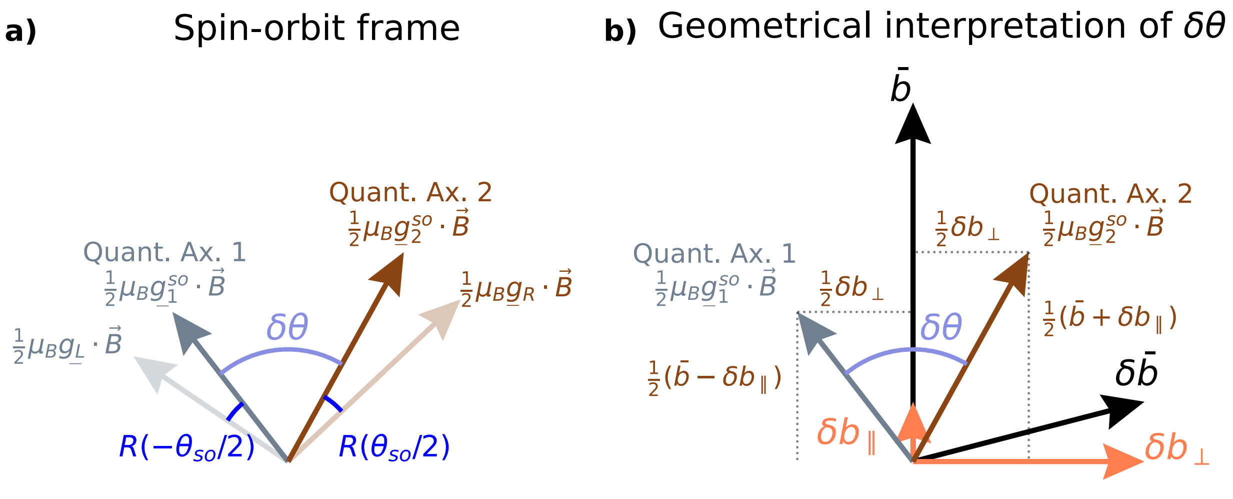

Supplementary Figure 2. **Schematics of geomtetrical interpretation** (a) Schematic explaining the difference between the laboratory and the spin-orbit frames. The light grey/brown arrows correspond to the g-tensors of left/right quantum dots, respectively. The dark grey/brown arrows represent the quantization axes in the spin-orbit frame, where the light quantization axes have been rotated by an angle $\theta_{\mathrm{so}}/2$ in order to take into account the spin-flip tunneling. The angle $\delta\theta$ between quantization axes 1 and 2 considers the spin-flip tunneling and the tilt between the g-tensors $\underline{g}_L$ and $\underline{g}_R$. (b) Geometrical explanation of $\delta\theta$ using the parameters extracted from the spectroscopy as a function of the magnetic field. By applying the trigonometric relations one can prove that $\delta\theta = \arctan\left[\delta g_\perp/(\bar{g}+\delta g_\parallel)\right] + \arctan\left[\delta g_\perp/(\bar{g}-\delta g_\parallel)\right]$.

SUPPLEMENTARY NOTE 3. RABI CHEVRONS AND GATE FIDELITIES

Here we show Rabi chevrons of the three studied transitions in the in-plane magnetic field direction and the estimation of the gate fidelities.

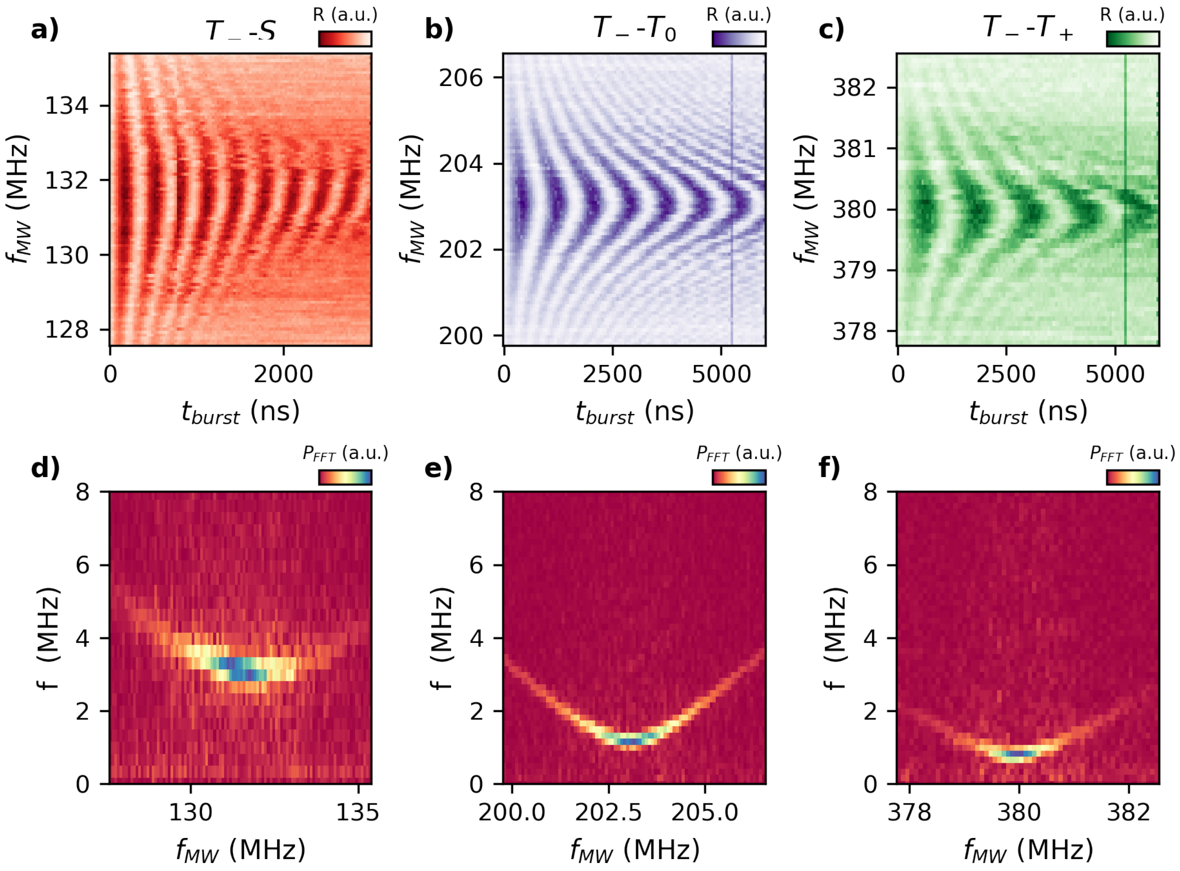

Supplementary Figure 3. (a-c) Rabi chevron for the 3 transitions at B_y = 40 mT and ε = 4.0 meV. (d-f) Fast Fourier transformation for the Rabi Chevrons. The distinction between the T_- and T_+ states is made possible by the Landau-Zener transition probability when returning to the readout point. In our system, the T_- state in the effective (1,1) configuration maps into an unblocked state due to the anticrossing, while the other states remain blocked.

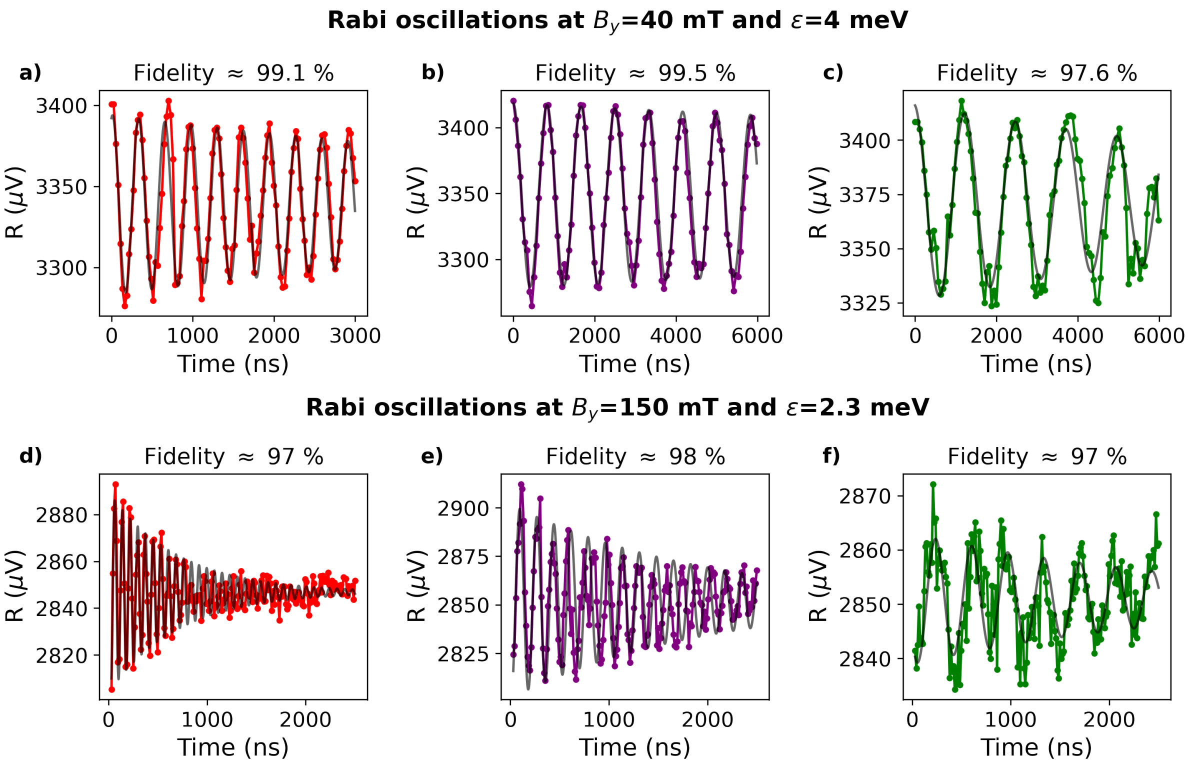

Supplementary Figure 4. Rabi oscillations for the three transitions investigated at different magnetic field and detuning point. Each trace is fitted to: $A\cos(2\pi f_R t+\phi)\exp\left(-t/T_2^{\mathrm{Rabi}}\right)+B$. Data (a-c) are the resonant traces from the Rabi chevrons in Supplementary Fig.3. (d-f) are the line traces at 150 mT from the data shown in Supplementary Fig. 5. The gate fidelity of each transition is estimated using $F=1/2(1+\exp\left(-T_2^{\mathrm{Rabi}} f_R/2\right))$, where T_2^{Rabi} the Rabi decay and f_R the Rabi frequency [1].

SUPPLEMENTARY NOTE 4. RAW DATA OF RABI OSCILLATION DEPENDENCES

In this section, we present the raw data of the measured Rabi oscillations as a function of magnetic field and detuning. For each measurement, the corresponding fast Fourier transform is shown below, revealing the dependence of the Rabi frequency. We also show an extra dataset of Rabi frequencies for the three transitions as a function of detuning and magnetic field in Supplementary Fig. 8. These plots were measured using a different magnetic field and detuning configuration compared to Fig.3 in the main text, demonstrating that the model works for different parameters.

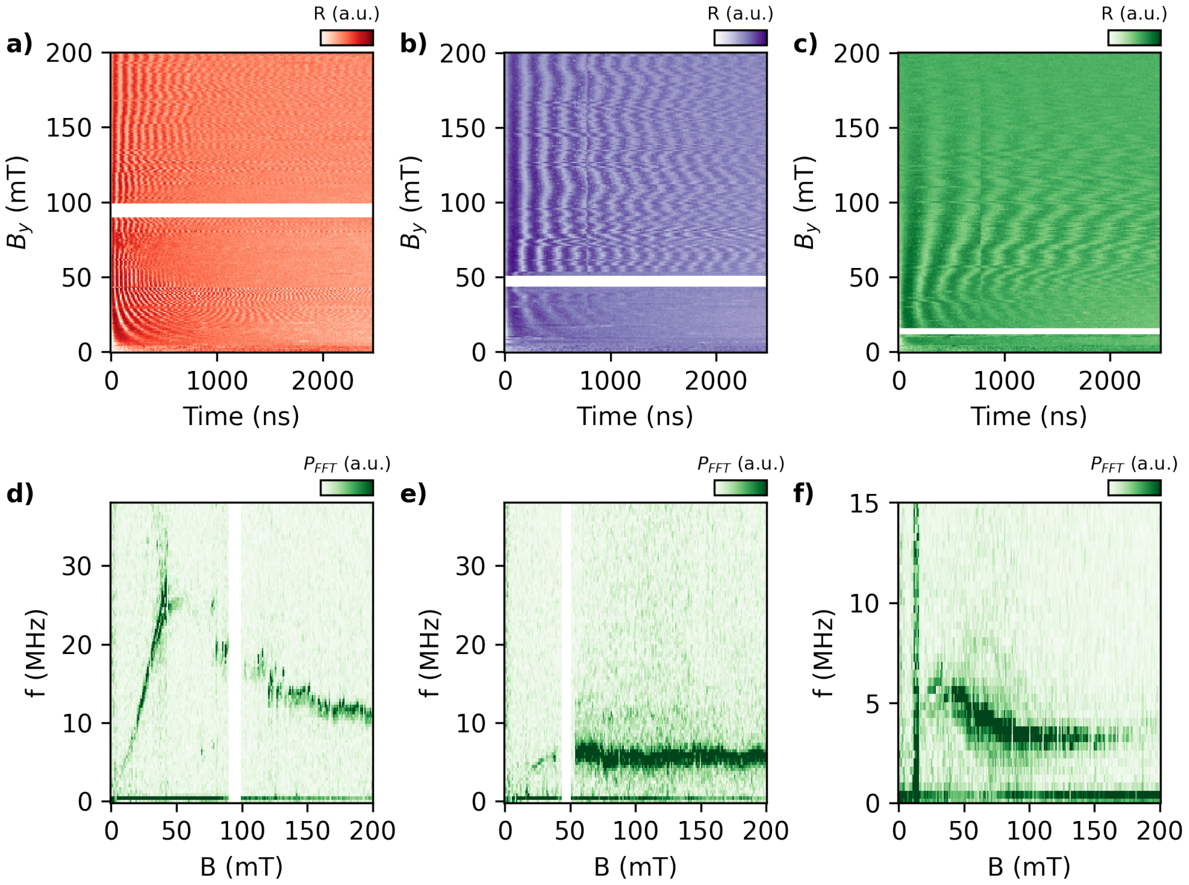

Supplementary Figure 5. Panels (a-c) show Rabi oscillations at $\varepsilon = 2.34$ meV as a function of magnetic field B_y for the three transitions. Panels (d-f) show the fast Fourier transform (FFT) of the top panels. The magnetic field-independent signal observed at low frequencies (below 1 MHz) is an artifact of the FFT, resulting from the decay of the Rabi oscillations.

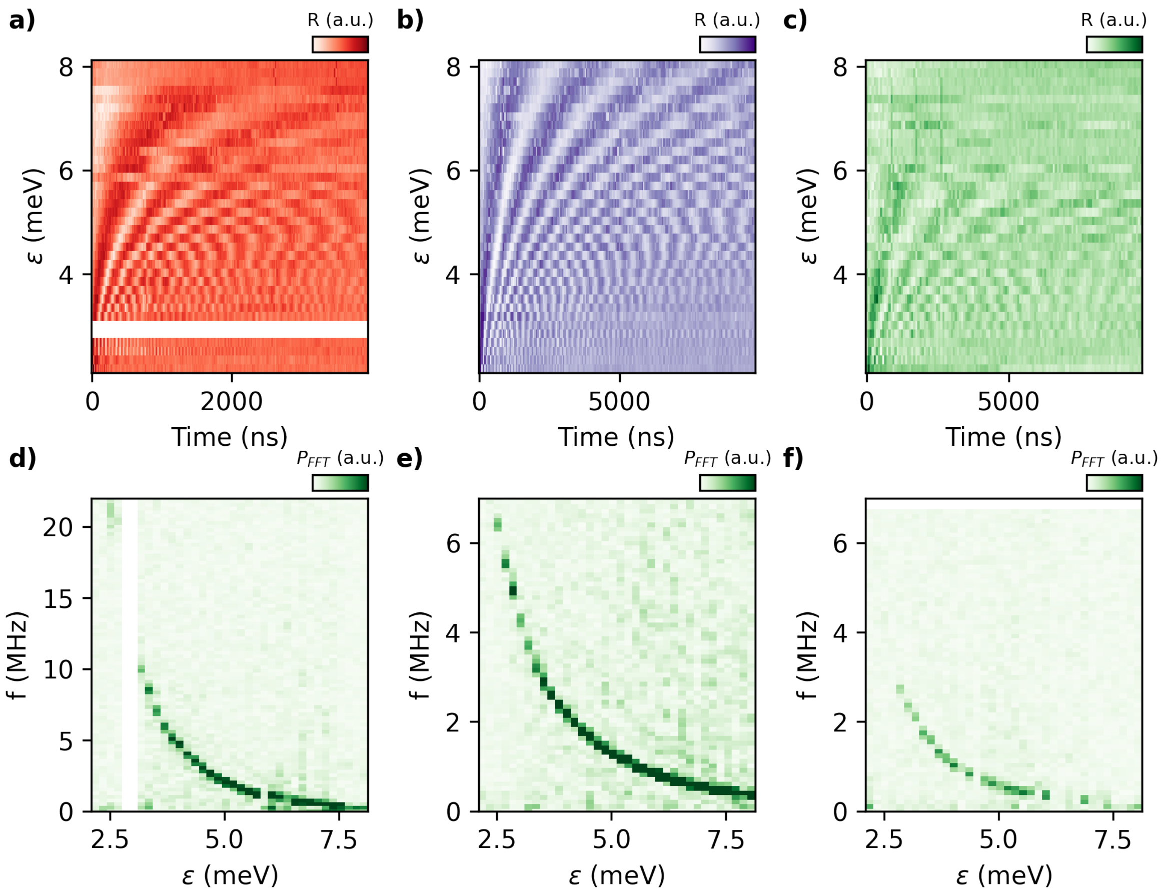

Supplementary Figure 6. Panels (a-c) show Rabi oscillations at $B_y = 90$ mT as a function of ε for the three transitions. Panels (d-f) show the fast Fourier transform (FFT) of the top panels.

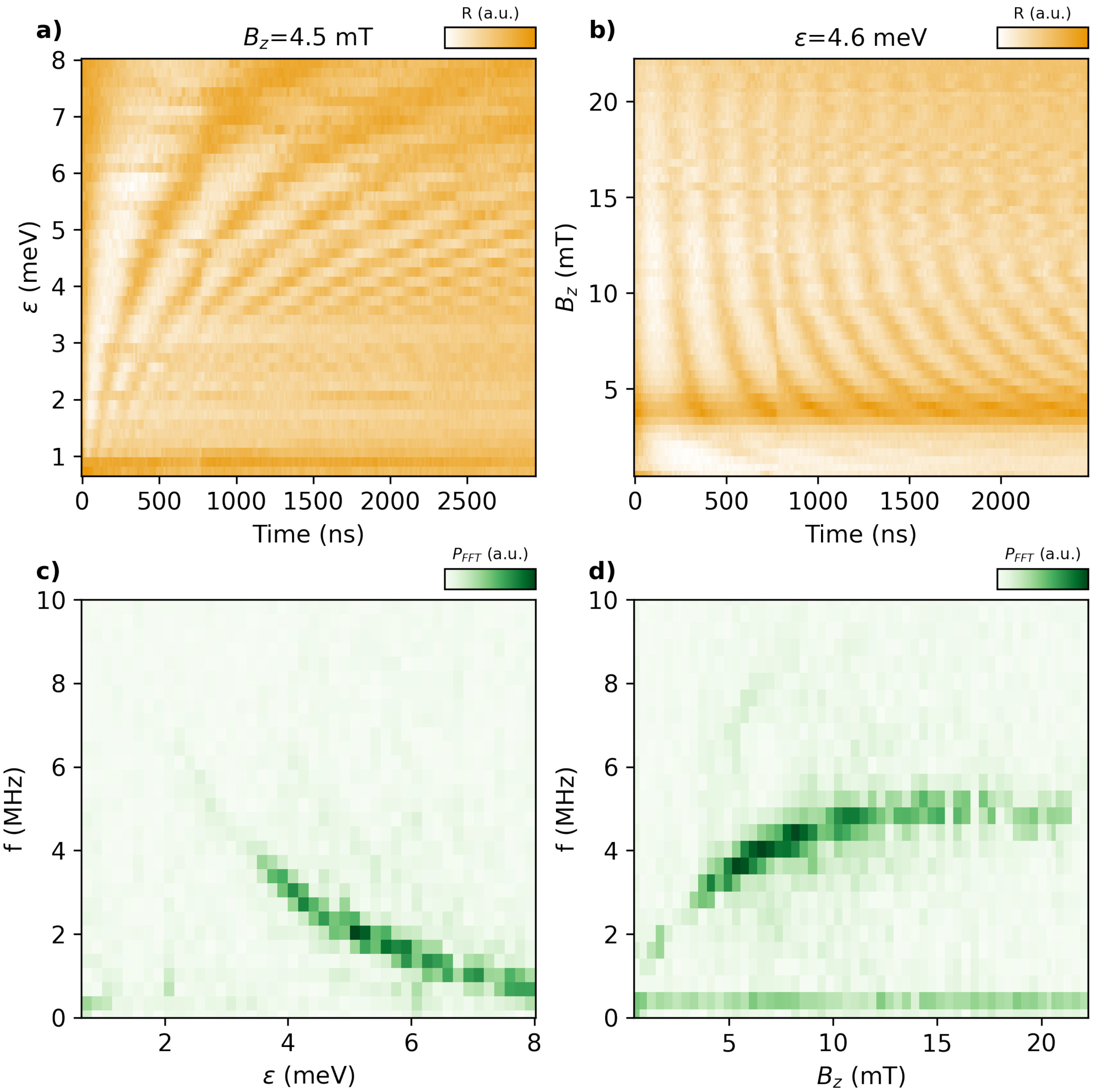

Supplementary Figure 7. Panels a) and b) show Rabi oscillations out-of-plane as a function of detuning and magnetic field, respectively. Panels c) and d) show the respective fast Fourier transforms of the top panels. This is the data used for Fig.4(c) and (d).

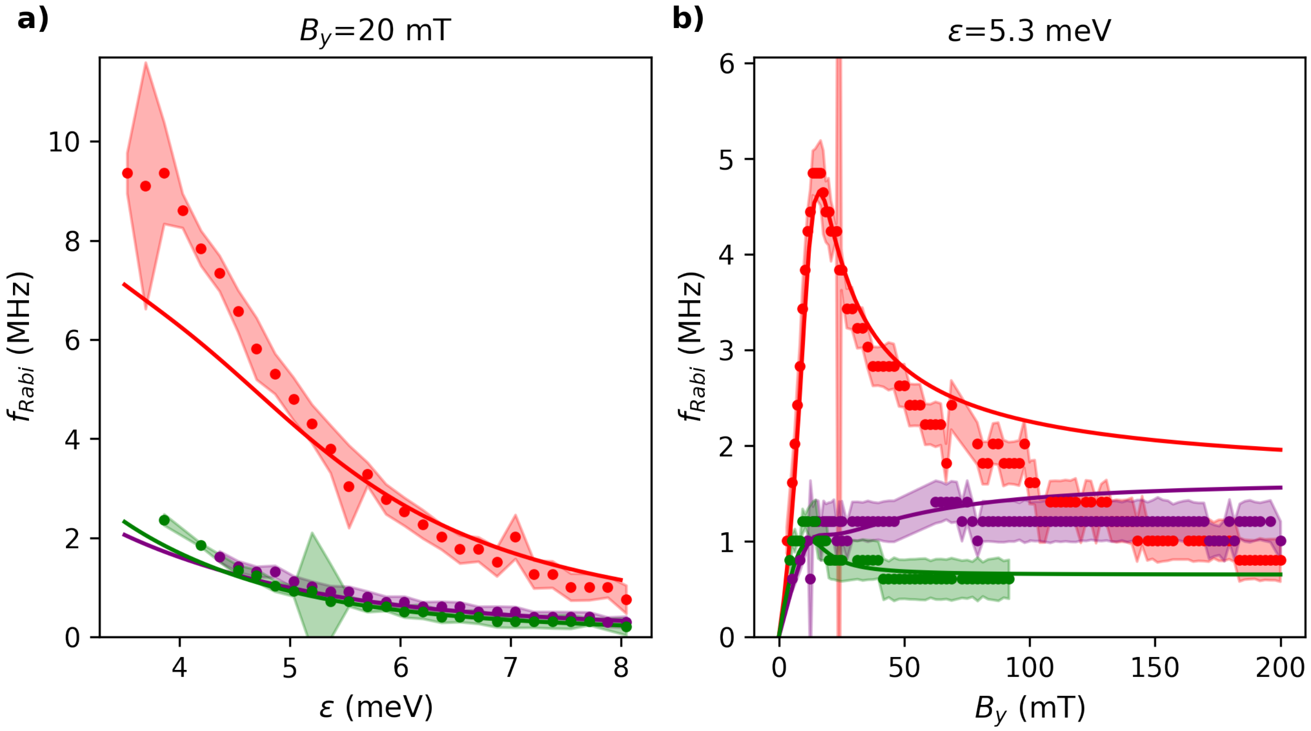

Supplementary Figure 8. Rabi frequencies of the three transitions as a function of detuning (a) and magnetic field (b). The exact solution for the Rabi frequencies is shown with solid lines. These plots have been measured for a different magnetic field and detuning configuration compared with Fig.3 of the main text.

SUPPLEMENTARY NOTE 5. LEVER ARMS AND CROSS-CAPACITANCE OF GATE BR

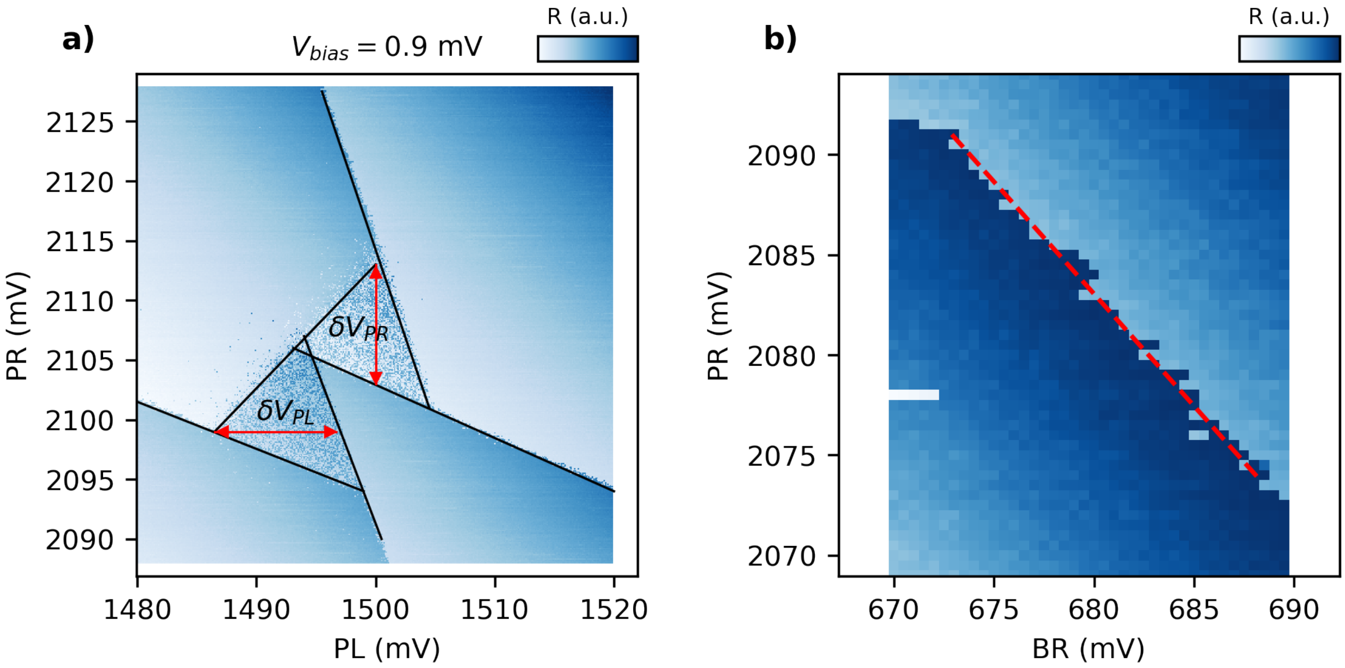

Supplementary Figure 9. a) Bias triangles at a bias voltage of 0.9 mV measured in reflectometry. From $\delta V_{PL} = 11.7$ mV and $\delta V_{PR} = 10.8$ mV we extract the left (right) lever arm $\alpha_{PL} = 0.083$ eV/V ($\alpha_{PR} = 0.077$ eV/V). b) Shift of the electrochemical potential in the right quantum dot due to the cross-capacitance from the barrier right (BR). From the slope, indicated with the dashed line, we extract a cross-capacitance $\gamma_{BR-PR} = 1.1$, which implies that BR has a stronger effect on the electrochemical potential of the right QD than the plunger gate. From α_{PR} and γ_{BR-PR} we estimate the effect of the microwave burst applied on BR has on the detuning.

SUPPLEMENTARY NOTE 6. EXTRA OUT-OF-PLANE DATA

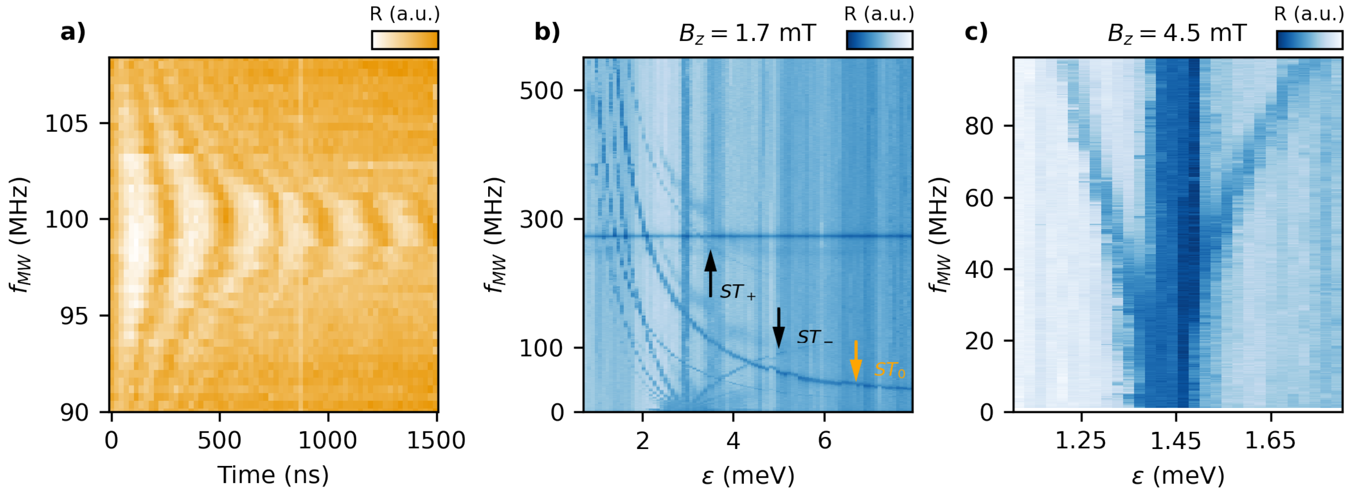

Supplementary Figure 10. Additional measurements in the out-of-plane direction at 7 mT and $\varepsilon = 4.0$ meV. a) Rabi Chevron showing microwave driven $S - T_0$ oscillations. b) Spectroscopy revealing the evolution of the three transitions with detuning. c) Zoom-in at low powers of the crossing between the S and T_- states. The change in the background signal at 1.45 meV does not allow resolving the merging point of the two lines and therefore whether a crossing or an anticrossing takes place, but it allows to set the upper bound for the anticrossing size at 30 MHz. The fact that there is a blocked return probability at 1.45 meV demonstrates that there is an anticrossing where the S and T_- states mix.

SUPPLEMENTARY NOTE 7. SUBHARMONIC PROCESSES

In this section, we provide additional data shining light into the origin of the integer fractions of the Larmor frequency, also referred as the subharmonic transitions. Such transitions have been also characterized in Refs. [2, 3]. Panel (a) presents the spectrum at $B_y = 70$ mT as a function of the microwave power. At low power levels, only the $T_- - S$ and $T_- - T_0$ transitions are observed (indicated by solid red and purple lines, respectively). As the power increases, the $T_- - T_+$ transition emerges, followed by subharmonics of the other two transitions (dashed lines). Panel (b) shows the same dependence but at a lower detuning, corresponding to a larger exchange interaction. Here, due to the highly nonlinear behavior of J at such low detuning, subharmonic effects appear at lower powers, allowing the observation of more transitions. We next focus on studying the $T_- - T_0$ transition for the fundamental frequency and its first subharmonic, also referred to in the literature as "one-photon" and "two-photon" processes. Panels (c) and (d) demonstrate that the $T_- - T_0$ transition can be coherently driven by both one-photon and two-photon processes. By analyzing the power dependence of these transitions, we find that the Rabi frequencies follow a power-law relationship, where the exponent corresponds to the number of photons involved in the process, as previously reported in [3].

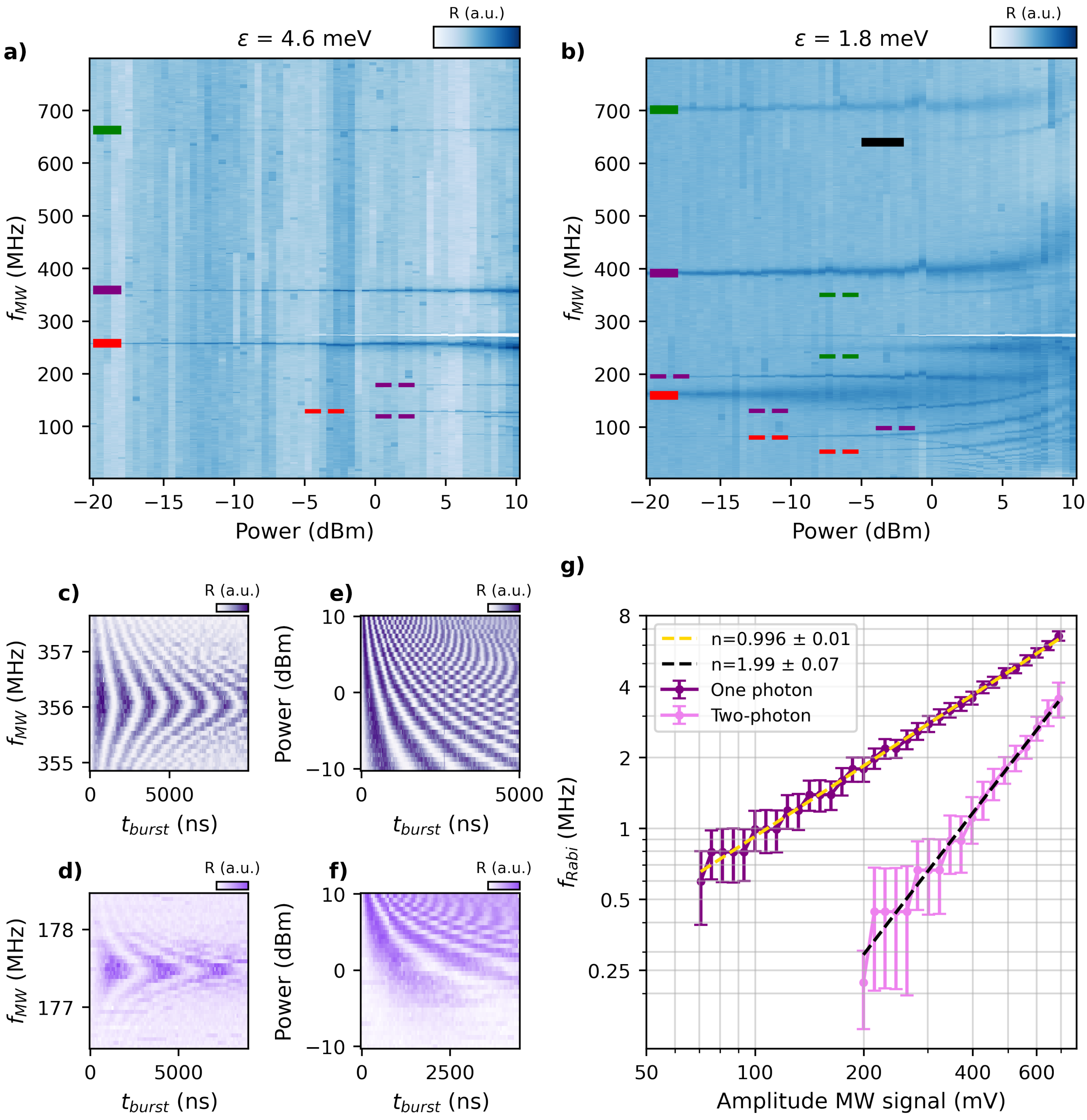

Supplementary Figure 11. Spin transitions as a function of the burst power at $B_y = 70$ mT for a detuning of 4.6 meV in (a) and 1.8 meV (b). Solid coloured lines indicate the larmor frequency of the transitions and dashed coloured lines the subharmonic transitions. The black solid line indicates a transition which only appears at low detunings and high powers. (c) Rabi Chevron of transition $T_- - T_0$ driven at the Larmor frequency. (d) Rabi Chevron of transition $T_- - T_0$ driven at the half value Larmor frequency ("two-photon process"). Power dependence of Rabi oscillations driven by (e) "one-photon" process and (f) "two-photon" process. (g) Fast Fourier Transform of (e) and (f) showing the Rabi frequency dependence as a function of the amplitude of the microwave signal.

SUPPLEMENTARY NOTE 8. NOISE MODEL AND FITTING PARAMETERS

Following the noise model introduced in reference [4], the dephasing time is:

$$\frac{1}{T_2^*} = \frac{\sqrt{\langle(\delta E)^2\rangle}}{\sqrt{2}\hbar} \tag{1}$$

where δE are the energy fluctuations of each transition. These fluctuations can be written as:

$$\delta E = \delta\varepsilon \frac{dJ}{d\varepsilon}\frac{dE}{dJ} + \delta E_{\Delta Z}\frac{dE}{d(\delta b_\perp)} + \delta E_Z \frac{dE}{d\bar{b}} \tag{2}$$

where $\delta\varepsilon$ are the fluctuations in detuning, $\delta E_{\Delta Z}$ are the fluctuations on $b_\perp$ and δE_Z the fluctuations of the total Zeeman energy.

By squaring δE we can express the terms in as root-mean-square (r.m.s.):

$$\langle \delta E^2 \rangle = \delta\varepsilon_{rms}^2 \left(\frac{dJ}{d\varepsilon}\frac{dE}{dJ}\right)^2 + \delta E_{\Delta Z_{rms}}^2 \left(\frac{dE}{d(\delta b_\perp)}\right)^2 + \delta E_{Z_{rms}}^2 \left(\frac{dE}{d\bar{b}}\right)^2 \tag{3}$$

With the analytical formulas presented in Methods, one can obtain $\langle \delta E^2 \rangle$ for the measured transitions.

B field direction	Transition	$\delta\varepsilon_{rms}$ (μeV)	$\delta E_{Z_{rms}}$ (neV)	$\delta E_{\Delta Z_{rms}}$ (neV)
In-plane	$T_- - S$	42.5 ± 5.5	1.3 ± 0.2	0.2 ± 0.2
In-plane	$T_- - T_0$	38.1 ± 3.5	0.3 ± 0.2	0.1 ± 0.5
In-plane	$T_- - T_+$	37.1 ± 3.7	0.0 ± 0.1	0.1 ± 0.6
Out-of-plane	$S - T_0$	36.0 ± 2.0	-	3.3 ± 0.2

Supplementary Table I. Summary of $\delta\varepsilon_{rms}$, $\delta E_{Z_{rms}}$, and $\delta E_{\Delta Z_{rms}}$ for the fits in Fig.5(a) for $T_- - S$, $T_- - T_0$ and $T_- - T_+$, and from Fig.5(c) for $S - T_0$. It is important to note that $\delta E_{\Delta Z_{rms}}$ and $\delta E_{Z_{rms}}$ are influenced by both magnetic field noise via hyperfine interactions and charge noise via g-factors.

SUPPLEMENTARY NOTE 9. FITS OF RAMSEY OSCILLATIONS

Here we present the measured Ramsey oscillations from which we extract the inhomogeneous dephasing time T_2^* by fitting the data to the expression $\mathrm{A}\cos(\omega t)e^{-t/T_2^*} + \mathrm{B}$. In this section we present the measurements and the fits from Figure 5.

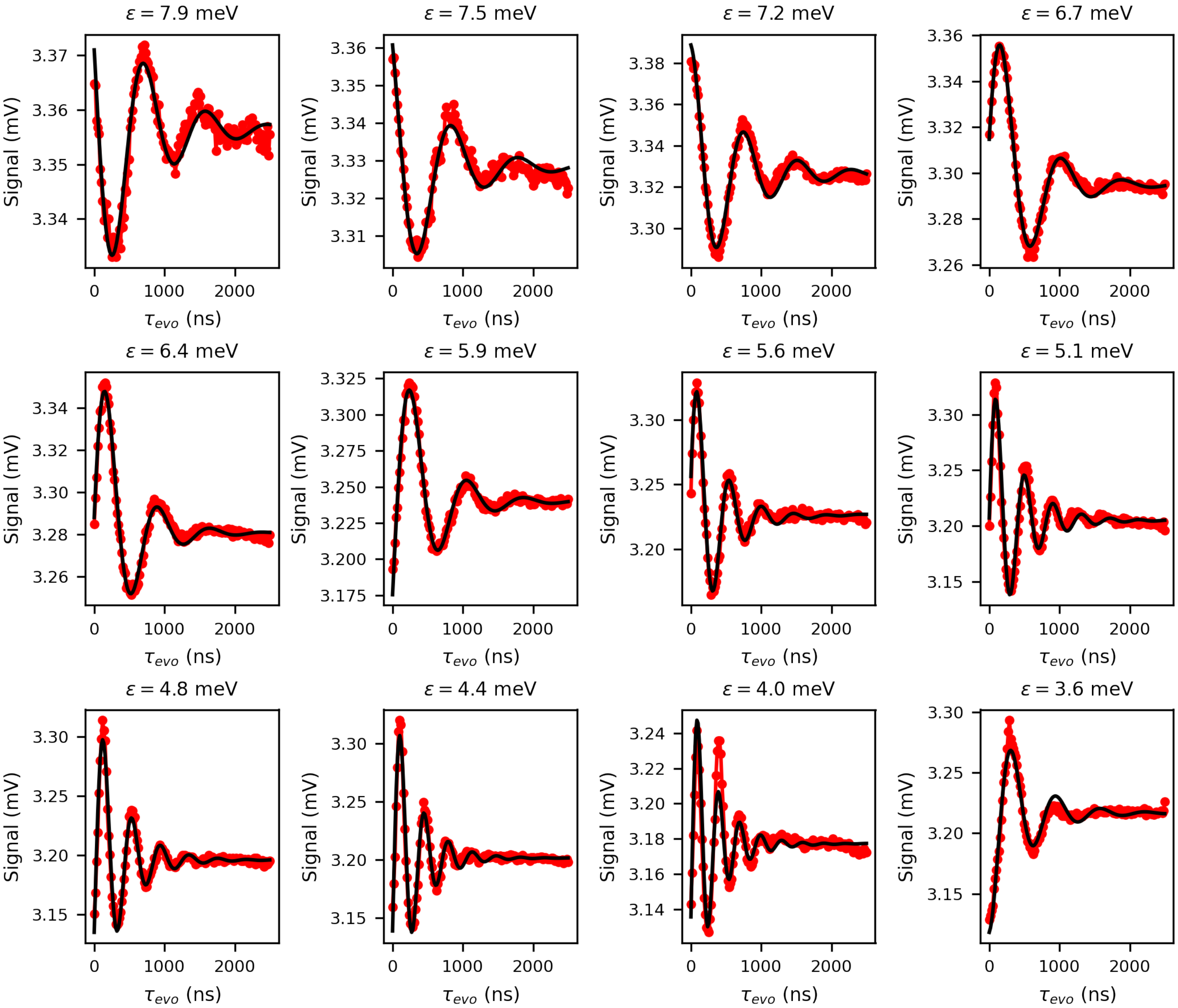

Supplementary Figure 12. Ramsey oscillations at $B_y = 30$ mT for the $T_- - S$ transition. Each trace is taken at a different detuning point and the total integration time is 20 minutes. Solid lines are the fits to $\mathrm{A}\cos(\omega t)e^{-t/T_2^*} + \mathrm{B}$. Each of these points is represented in Fig.5(a)

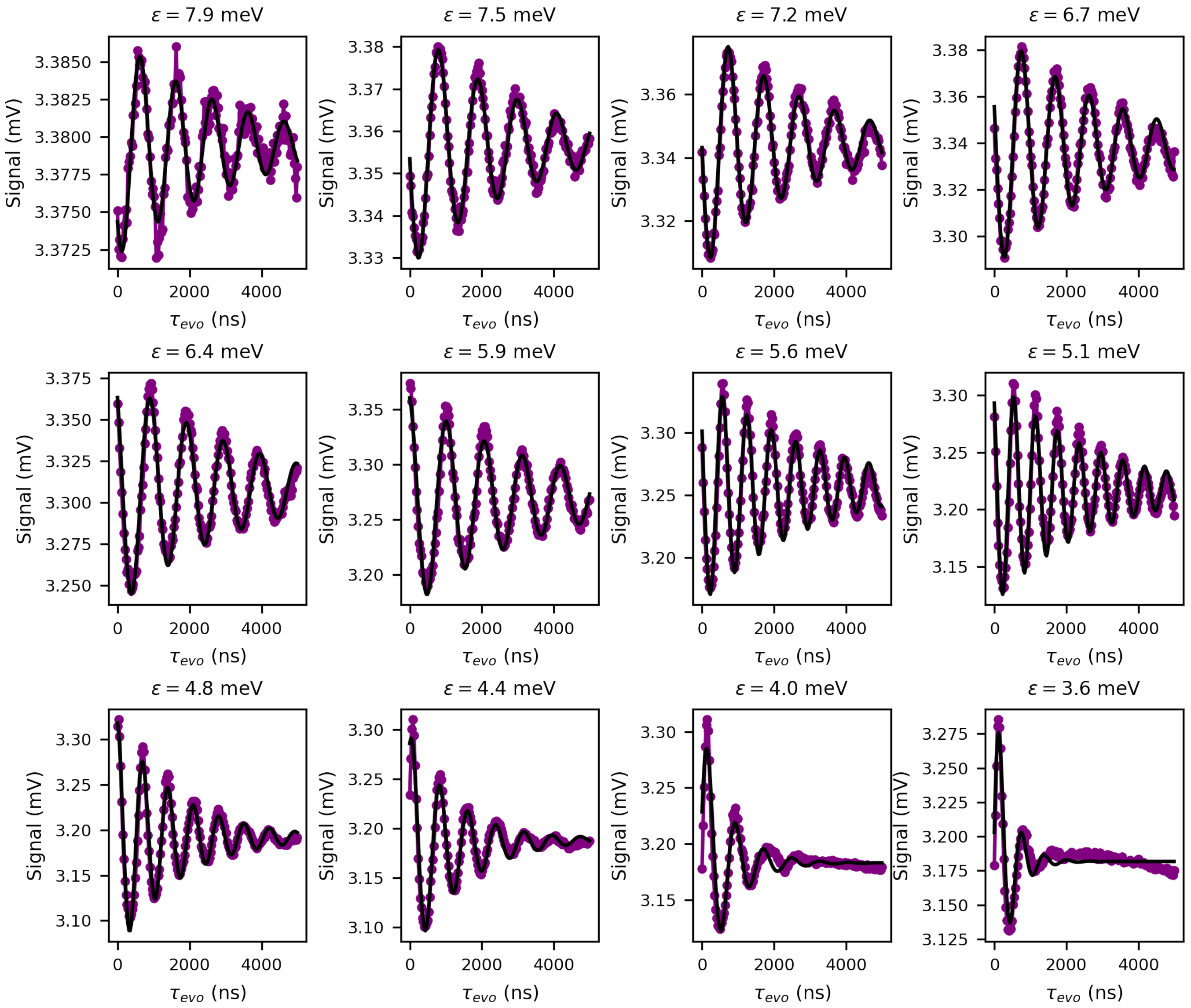

Supplementary Figure 13. Ramsey oscillations at $B_y = 30$ mT for the $T_- - T_0$ transition. Each trace is taken at a different detuning point and the total integration time is 20 minutes. Solid lines are the fits to A$\cos(\omega t)e^{-t/T_2^*}$ + B. Each of this points is represented in Fig.5(a)

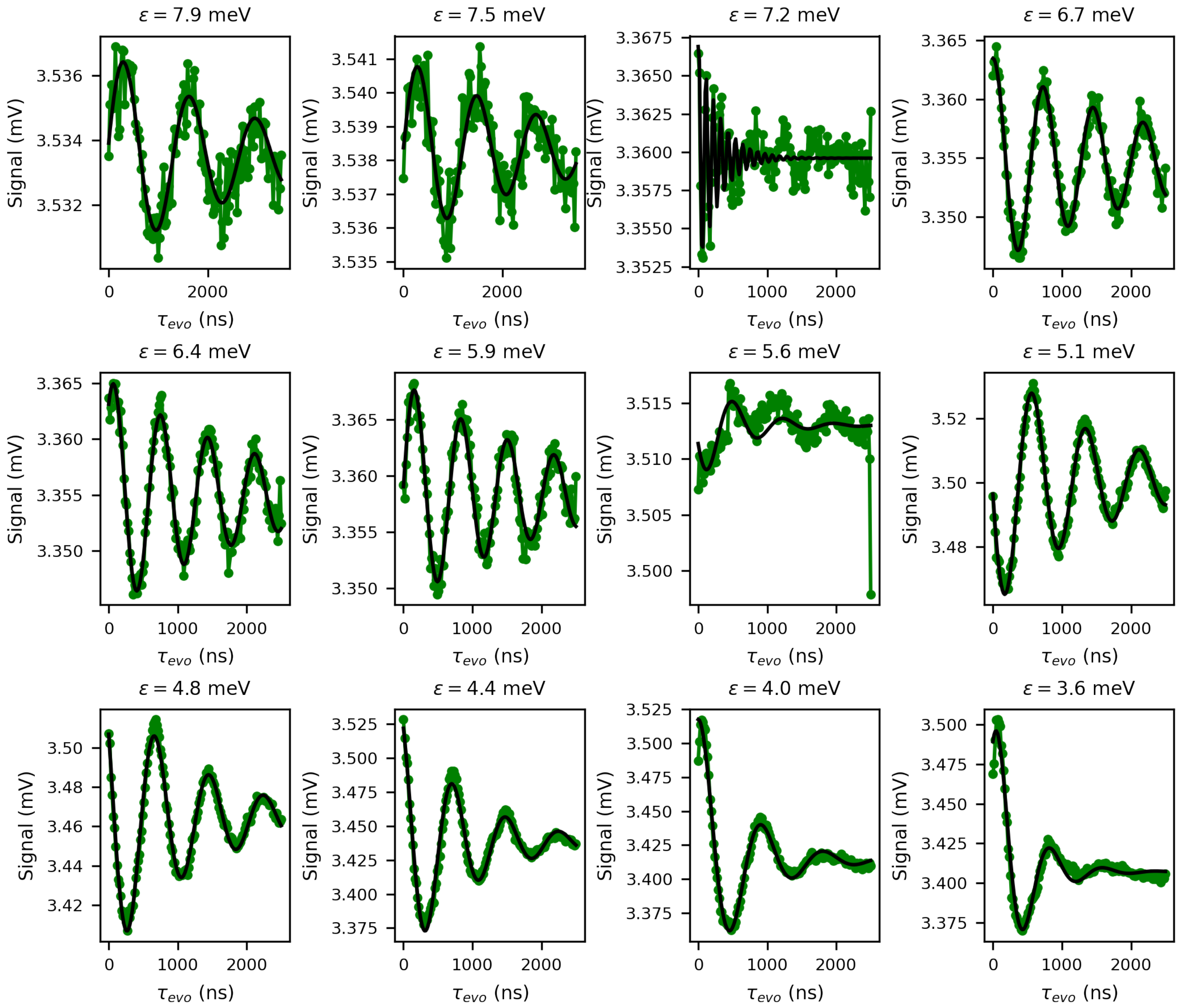

Supplementary Figure 14. Ramsey oscillations at $B_y = 30$ mT for the $T_- - T_+$ transition. Each trace is taken at a different detuning point and the total integration time is 20 minutes. Solid lines are the fits to $\mathrm{A}\cos(\omega t)e^{-t/T_2^*} + \mathrm{B}$. Each of this points is represented in Fig.5(a). The panels at ε =7.2 meV and ε =5.6 meV correspond to the outliers in Fig.5(a).

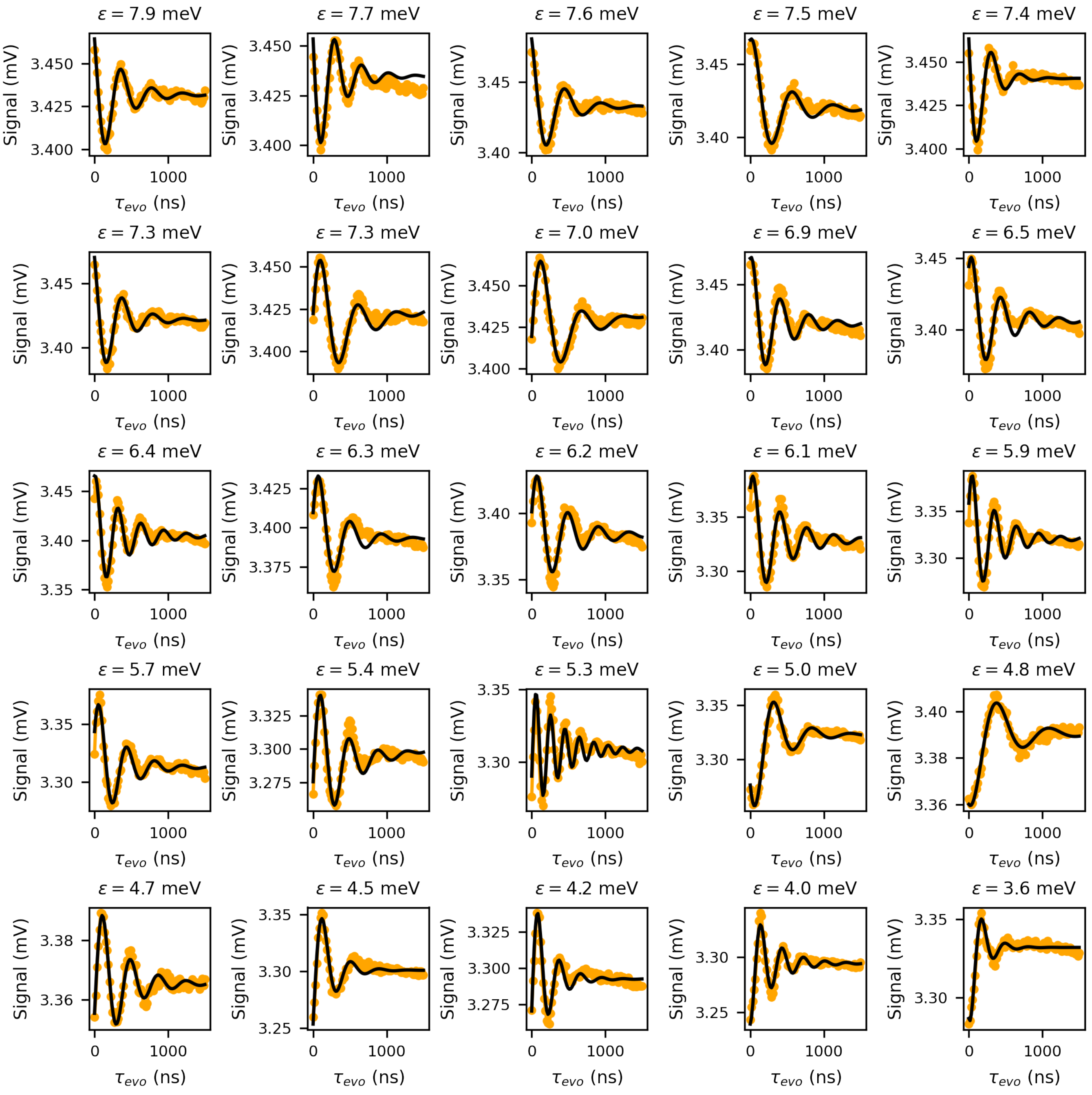

Supplementary Figure 15. Ramsey oscillations at $B_z = 12.5$ mT for the $S - T_0$ transition. Solid lines are the fits to $A\cos(\omega t)e^{-t/T_2^*} + B$. Each of this points is represented in Fig.5(c).

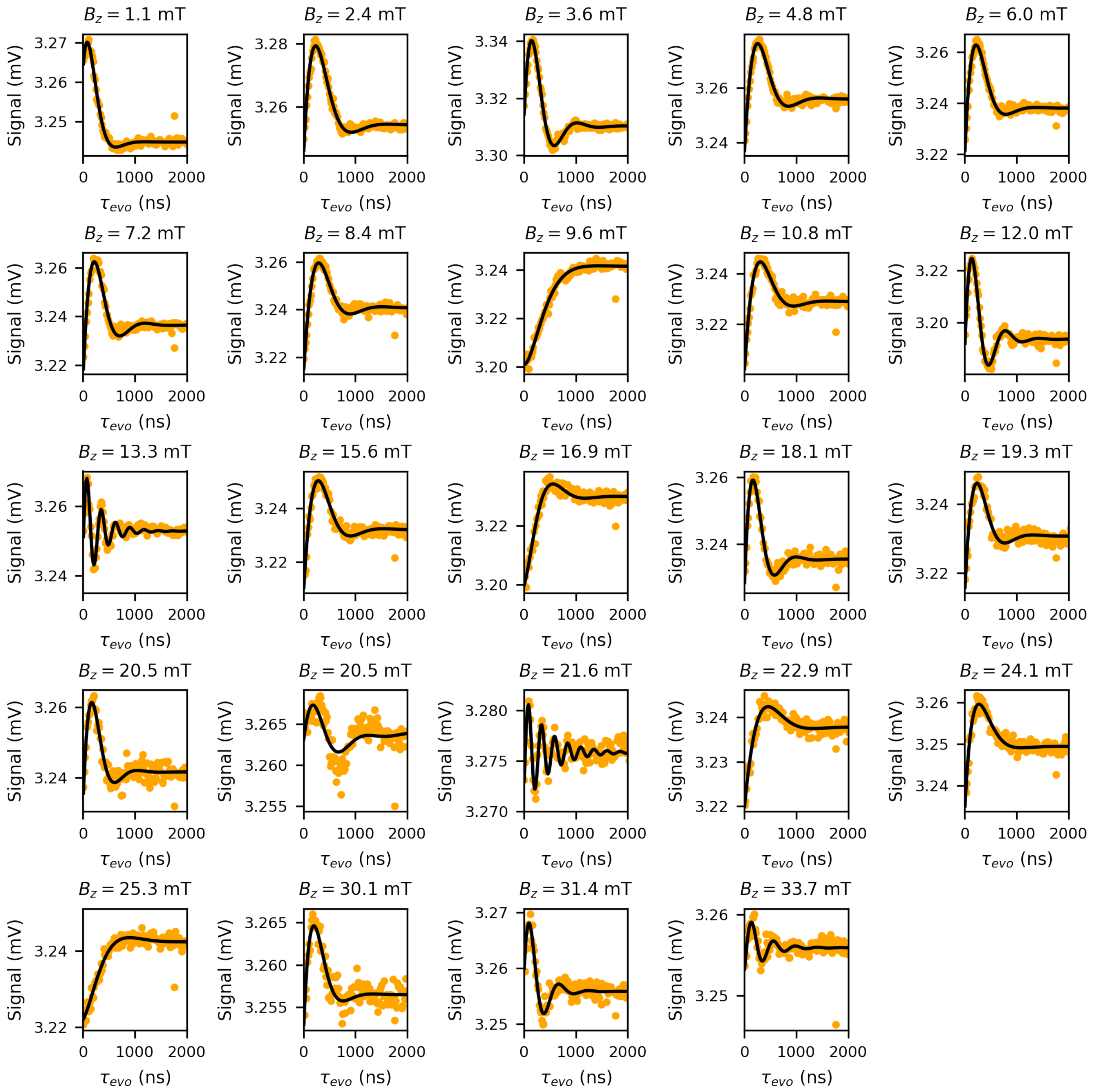

Supplementary Figure 16. Ramsey oscillations at $\varepsilon = 7.4$ meV for the $S - T_0$ transition. Solid lines are the fits to $\mathrm{A}\cos(\omega t)e^{-t/T_2^*} + \mathrm{B}$. Each of this points is represented in Fig.5(d).

REFERENCES

[1] Stano, P. & Loss, D. Review of performance metrics of spin qubits in gated semiconducting nanostructures. Nature Reviews Physics **4**, 672–688 (2022). URL https://www.nature.com/articles/s42254-022-00484-w. Publisher: Nature Publishing Group.

[2] Stehlik, J., Schroer, M., Maialle, M., Degani, M. & Petta, J. Extreme Harmonic Generation in Electrically Driven Spin Resonance. Physical Review Letters **112**, 227601 (2014). URL https://link.aps.org/doi/10.1103/PhysRevLett.112.227601. Publisher: American Physical Society.

[3] Scarlino, P. et al. Second-Harmonic Coherent Driving of a Spin Qubit in a Si/SiGe Quantum Dot. Physical Review Letters **115**, 106802 (2015). URL https://link.aps.org/doi/10.1103/PhysRevLett.115.106802.

[4] Dial, O. E. et al. Charge Noise Spectroscopy Using Coherent Exchange Oscillations in a Singlet-Triplet Qubit. Physical Review Letters **110**, 146804 (2013). URL https://link.aps.org/doi/10.1103/PhysRevLett.110.146804. Publisher: American Physical Society.
